# A Bioinspired Design of Protective Al_2_O_3_/Polyurethane Hierarchical Composite Film Through Layer‐By‐Layer Deposition

**DOI:** 10.1002/advs.202402940

**Published:** 2024-05-20

**Authors:** Jiaming Zhong, Zhixiong Wen, Yibo Wu, Hao Luo, Guodong Liu, Jianqiao Hu, Hengxu Song, Tao Wang, Xudong Liang, Helezi Zhou, Wei Huang, Huamin Zhou

**Affiliations:** ^1^ State Key Laboratory of Materials Processing and Die and Mould Technology School of Materials Science and Engineering Huazhong University of Science and Technology Wuhan 430074 China; ^2^ Luoyang Ship Material Research Institute Luoyang 471023 China; ^3^ LNM Institute of Mechanics Chinese Academy of Sciences Beijing 100190 China; ^4^ School of Engineering Science University of Chinese Academy of Sciences Beijing 100049 China; ^5^ National Key Laboratory of Explosion Science and Safety Protection Beijing Institute of Technology Beijing 100081 China; ^6^ School of Science Harbin Institute of Technology (Shenzhen) Shenzhen 518055 China

**Keywords:** bioinspired designs, energy absorption, impact resistance, nanocomposite, thin film

## Abstract

Structural materials such as ceramics, metals, and carbon fiber‐reinforced plastics (CFRP) are frequently threatened by large compressive and impact forces. Energy absorption layers, i.e., polyurethane and silicone foams with excellent damping properties, are applied on the surfaces of different substrates to absorb energy. However, the amount of energy dissipation and penetration resistance are limited in commercial polyurethane foams. Herein, a distinctive nacre‐like architecture design strategy is proposed by integrating hard porous ceramic frameworks and flexible polyurethane buffers to improve energy absorption and impact resistance. Experimental investigations reveal the bioinspired designs exhibit optimized hardness, strength, and modulus compared to that of polyurethane. Due to the multiscale energy dissipation mechanisms, the resulting normalized absorbed energy (≈8.557 MJ m^−3^) is ≈20 times higher than polyurethane foams under 50% quasi‐static compression. The bioinspired composites provide superior protection for structural materials (CFRP, glass, and steel), surpassing polyurethane films under impact loadings. It is shown CFRP coated with the designed materials can withstand more than ten impact loadings (in energy of 10 J) without obvious damage, which otherwise delaminates after a single impact. This biomimetic design strategy holds the potential to offer valuable insights for the development of lightweight, energy‐absorbent, and impact‐resistant materials.

## Graphical abstract

A multilayer structural design of energy absorption and impact‐resistant Al_2_O_3_/polyurethane composite thin film is proposed. Inspired by nacre and mantis shrimp dactyl clubs, efficient protection of various substrates from compressions and multiple impacts is realized because of the multiscale energy dissipation mechanisms. The resulting normalized absorbed energy (≈8.557 MJ m^−3^) under compression is ≈20 times higher than commercial polyurethane foams.

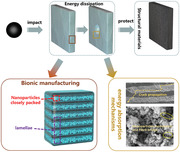

## 1 Introduction

Impact loadings pose significant threats to structural materials, particularly in the fields of defense, automobile, sports, and aerospace engineering.^[^ ^1^, ^2^, ^3^, ^4^, ^5^ ^]^ Fiber‐reinforced composites,^[^ ^6^, ^7^, ^8^ ^]^ as one of the most promising structural materials, have now been used in various fields owing to ingenious designability, large specific modulus, and high specific strength. However, catastrophic failure can occur in these materials serving in extreme environments.^[^ ^9^, ^10^, ^11^ ^]^ Damages like delamination and breakage of fibers accumulate during compressions and impacts, leading to severe property deterioration.^[^ ^12^, ^13^, ^14^ ^]^ Polyurethane and silicone foams have been used as energy absorption coatings on fiber‐reinforced composites to avoid catastrophic failure.^[^ ^15^, ^16^ ^]^ While the limited energy dissipation and resistance of penetration fall short of meeting application demands. Hence, it is crucial to explore more effective protection strategies for structural materials to avoid catastrophic failure under various loadings.

Nature offers abundant inspiration for developing energy‐dissipation materials.^[^ ^17^, ^18^, ^19^, ^20^, ^21^, ^22^, ^23^ ^]^ One representative example is the mollusk, which possesses both strong and tough shells to resist impact and compressive loadings from surroundings.^[^ ^24^, ^25^, ^26^ ^]^ The nacre layer of mollusk shells is typically brick‐and‐mortar lamellar architecture: the “bricks” are the mineral aragonite platelets, dispersed in an organic matrix (“mortar”). The organic matrix acts as a lubricant to allow adjacent “bricks” movement and limits excessive sliding between layers like glue.^[^ ^22^ ^]^ Moreover, it provides a path for crack deflection in further crack propagation to dissipate mechanical energy.^[^ ^20^ ^]^ On the other hand, mantis shrimps, as the predators of mollusks, have evolved superior dactyl clubs to break rigid mollusk shells. The secret of success in the performance of impact resistance is its hierarchical structure. Besides the well‐known herringbone‐like and Bouligand structural design of chitin fibers, researchers have recently paid attention to the close‐packed nanoparticles in the surface layer of dactyl clubs.^[^ ^27^, ^28^, ^29^ ^]^ This structural design dissipates a great deal of energy through processes such as nanoparticle translation, deformation, and pile‐up.^[^ ^30^, ^31^ ^]^ In addition, the modulus mismatch between nanoparticles and polymer matrix leads to the crack deflection and bridging of polymer fibrils across cracks, enhancing energy absorption.^[^ ^19^ ^]^

Inspired by the above characteristic structures, numerous attempts have been made to fabricate impact‐resistant materials.^[^ ^32^, ^33^, ^34^, ^35^, ^36^, ^37^, ^38^, ^39^ ^]^ For instance, a polyimide‐based nanocomposite film with a double‐layer nacre‐inspired structure showed enhanced mechanical properties, including strength, modulus, and hardness.^[^ ^40^ ^]^ Similarly, graphene oxide‐poly (methyl methacrylate) films with bionic multilayer laminate structures achieved optimized stiffness and strength.^[^ ^41^ ^]^ Additionally, a heterogeneous nacre‐like alumina‐poly (methyl methacrylate) composite integrated strength and fracture toughness, presenting superior impact energy absorption.^[^ ^42^ ^]^ The primary research in mimicking mantis shrimp focused on sinusoidal helicoidal and Bouligand structures. For example, a fiber composite laminate incorporating a sinusoidal helicoidal structure is obtained, enhancing impact resistance and damage tolerance, and drawing inspiration from the impact area of dactyl clubs.^[^ ^43^ ^]^ Overall, those previous reports have demonstrated that biomimetic structural design is an effective strategy for enhancing the mechanical properties of synthetic materials.

**Figure 1:**
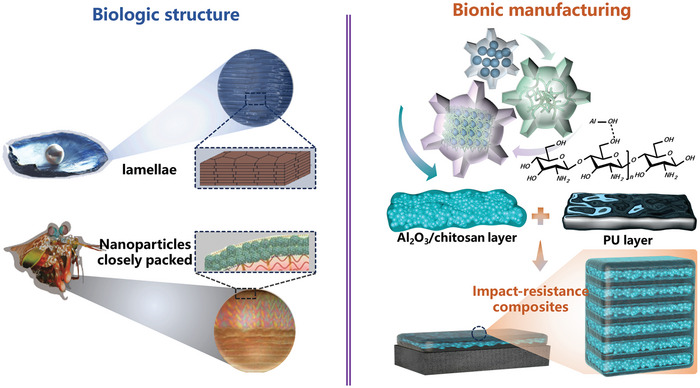
Schematic illustration of the structure design strategy and fabrication process of bionic APU composites. Blue dots, green curves, blue boxes with dots stacked, and black boxes represent Al_2_O_3_ nanoparticles, chitosan, hard layer, and soft layer, respectively. The microstructure of nacre and mantis shrimp were adapted with permission from.^[^ ^22^, ^27^, ^29^, ^30^ ^]^

In the present work, we proposed a distinctive architectural design strategy to fabricate energy absorption and impact resistance composite film, drawing inspiration from nacre and mantis shrimp (**Figure** **1**). With the aid of solvent evaporation‐induced self‐assembly, hard Al_2_O_3_ porous films with nanoparticles stacked structure were successfully fabricated. Al_2_O_3_/polyurethane hierarchical composites (APU) were then manufactured by hard Al_2_O_3_ porous film and soft polyurethane (PU) film laying layer‐by‐layer. Both quasi‐static compression and drop tower impacts indicated a significant increase in energy dissipation of bioinspired APU films compared to commercial PU films. Combined in‐depth characterization with finite element analysis (FEA), the multiscale energy absorption mechanisms were clarified. We demonstrated with the protection of the bioinspired composite film, structural materials such as carbon fiber reinforced composites (CFRP) can withstand multiple impacts without obvious damage, which otherwise can have severe delamination and fiber breakage after a single impact.

## 2 Composite Film with Bioinspired Hierarchical Structure

**Figure 2:**
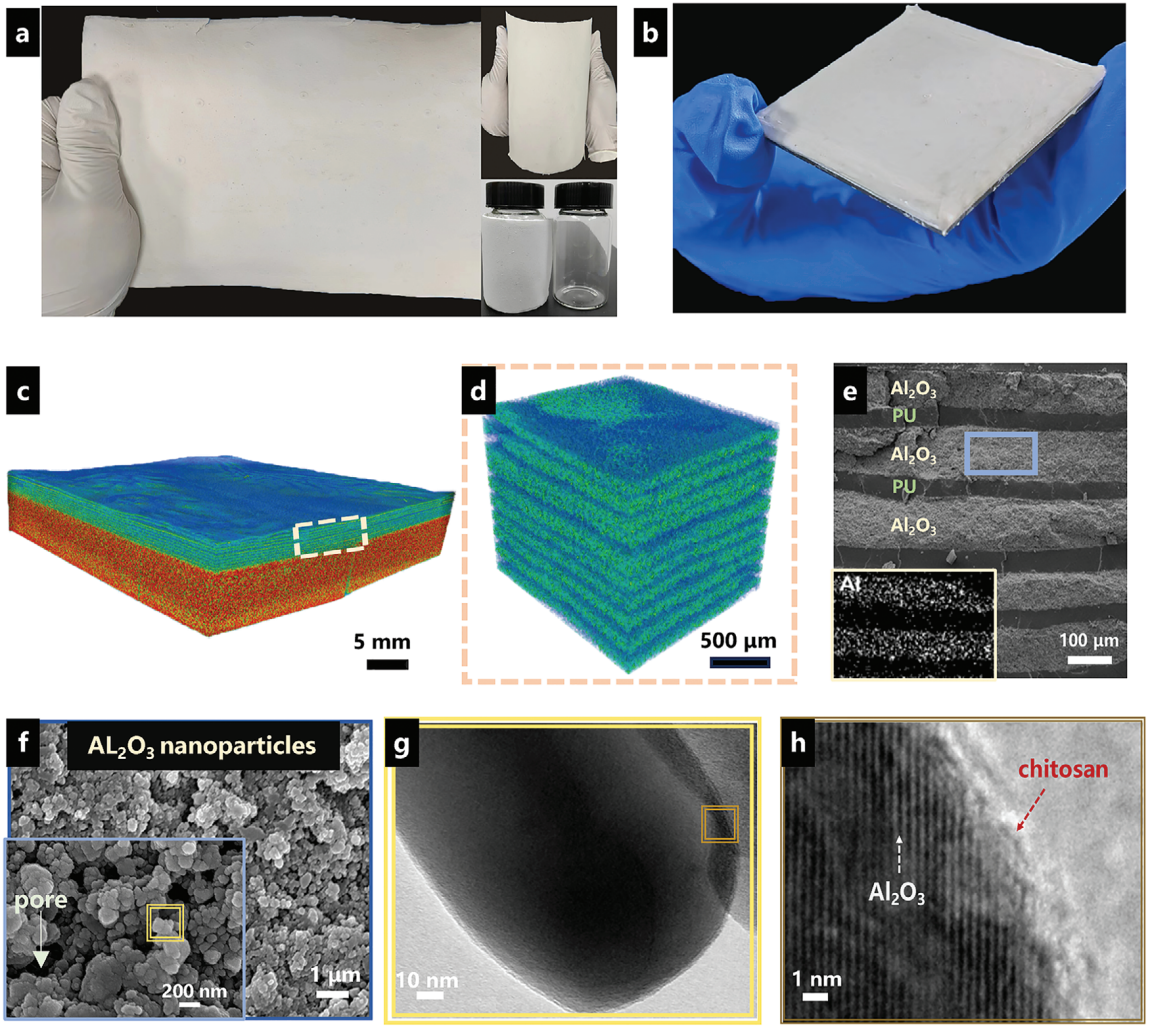
Multiscale structure of the bioinspired APU films. a) Digital photographs showing a large‐sized APU film. Inset images show the curved film, which can be applied to glass bottles. b) CFRP plate coated with APU film. c,d) Micro‐CT image representing the 3D reconstruction models. Layered structures with alternative densities are shown. e) Cross–sectional SEM and EDX image showing the nacre‐like lamellar architecture. The distribution of Al_2_O_3_ and PU is indicated. f) High‐resolution SEM images revealing the Al_2_O_3_ nanoparticles stacked in the thick layers with nanopores inside. g, h) High‐resolution TEM images showing the interfaces between nanoparticles and chitosan macromolecules attached to the particle surfaces.

The bioinspired APU films with delicate lamellar microstructure were fabricated via evaporation‐induced self‐assembly and spin/scratch coating methods. An as‐fabricated large‐sized APU film is shown in **Figure** **2**a. The inner photographs revealed the excellent flexibility of APU film, which could be applied to curved surfaces, such as glass bottles. The APU films could be applied on different substrates, including metals, glasses, and carbon fiber‐reinforced plastics (CFRP). Figure 2b presents a CFRP sample coated with APU film. When taking a further look at the microstructures with micro‐CT, the APU film on a glass substrate (Figure 2c,d) showed nacre‐like lamellar structures with alternating thick and thin layers. Scanning electron microscopy (SEM) and transmission electron microscopy (TEM) were then applied to analyze the details of the microstructures. The thickness of thick layers (≈100 µm) is ≈10 times that of thin layers (Figure 2e). The energy dispersive spectroscopy (EDX) under SEM (inset) showed the distribution of Al in APU film, consistent with the thick layer, which was formed by stacked Al_2_O_3_ nanoparticles in a chitosan network. Note that the mass fraction of Al_2_O_3_ nanoparticles is nearly 90 wt.% in the hard layer, proven by the thermogravimetric analysis (TGA) test (Figure S1, Supporting Information). According to the magnified SEM image (Figure 2f), the thick layer consisted of stacking nanoparticles, similar to the stacking nanoparticles structure of mantis shrimps.

Moreover, nanoscale pores existed in the Al_2_O_3_ thick layer due to the evaporation of solvent during the fabrication process (Figure 2f, inserted image), facilitating nanoparticle mobility and layer densification during compressions and impacts. High‐resolution TEM analysis (Figure 2g,h) revealed chitosan adjacent to Al_2_O_3_ nanoparticles, which suggested the chitosan builds the interacting network between nanoparticles. The effectiveness of protection provided by these bioinspired APU composites was further evaluated via quasi‐static compressions and drop‐tower impact tests.

### 2.1 Energy Absorption Under Quasi‐Static Loadings

**Figure 3:**
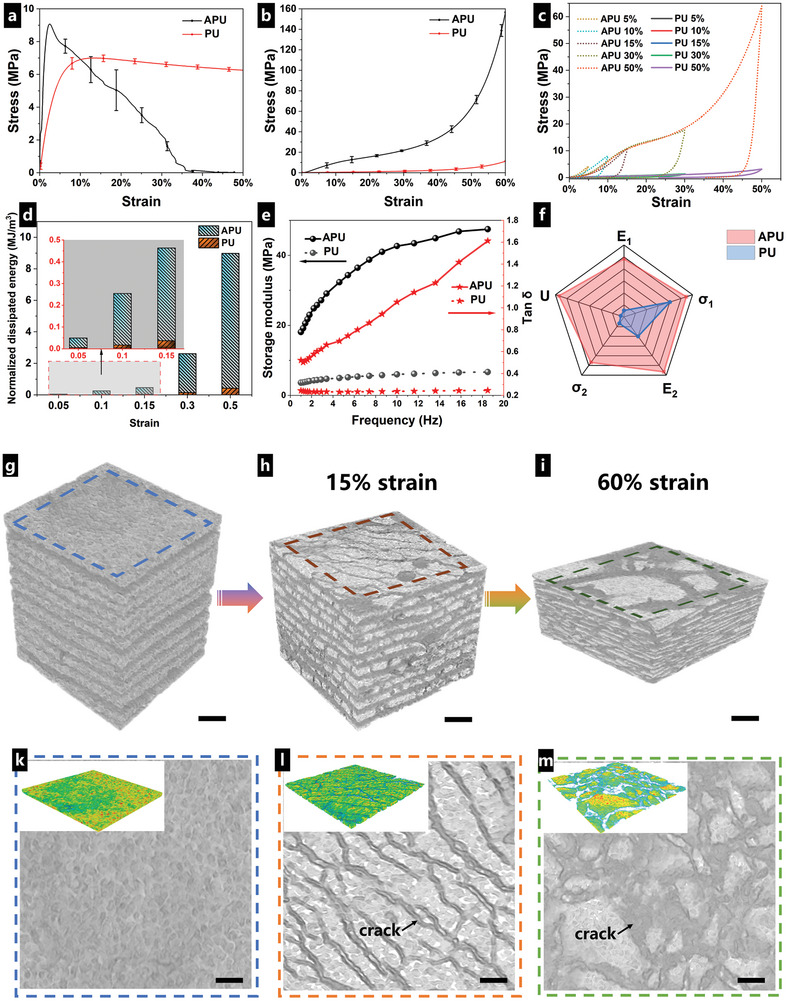
Investigation of quasi‐static mechanical properties of APU composites and PU. a) Tensile stress‐strain curves. b) Compression stress‐strain curves. c) Stress‐strain curves of compression‐recovery experiments with compression ratios of 5%, 10%, 15%, 30%, and 50%, respectively. d) The normalized dissipated energy during compression‐recovery tests. e) Plot of the storage modulus and tan δ as a function of dynamic frequency. f) Rader plots comparing the performance of APU composites and PU (*E*_1_, *E*_2_, *σ*_1_, *σ*_2,_ and *U* stand for tensile modulus, compression modulus, tensile strength, compression strength, and normalized dissipated energy, respectively). g–m) Micro‐CT images of APU film, APU film after a 15% compression, and APU film after a 50% compression with the scale bar of 500 µm.

The energy absorption properties of the bioinspired APU composite films were first evaluated under quasi‐static loading conditions. The optimized APU films with stacking nanoparticles and lamellar structure exhibited higher surface hardness, strength, and modulus compared to PU films. As shown in Figure S2 (Supporting Information), the shore hardness increased from ≈20 to 50D when replacing PU coating with bioinspired APU coatings on the CFRP. The resulting tensile modulus of APU composites was ≈484.7 MPa and nearly nine times enhancement compared to that of PU films (**Figure** **3**a). The tensile strength of APU was 9.1 MPa, increasing 31.9% compared to pure PU. When it came to the compression tests (Figure 3b), three distinct regions of elastic, yield, and hardening occurred in both types of samples. The stress of PU (red curve in Figure 3b) increased gradually and linearly with enhanced strain (< 40% strain), showing an elastic modulus of 6.8 MPa. A large deformation was presented with low stress, similar to hyperelastic rubber. After yield and hardening, the modulus was observed to reach ≈200 MPa. While in APU composites (black curve in Figure 3b), a substantial enhancement occurred in both strength and modulus. In the elastic region, the elastic modulus reached 52.2 MPa which was 7.7 times than that of PU films. Unlike the PU film, the yield stage occupied the majority of the compression curve of APU films. After that, the strain‐hardening behavior resulted in a modulus of 565 MPa and a strength of 156 MPa, which was far superior to PU films.

Compression‐recovery and dynamic mechanical analysis (DMA) experiments were performed to further investigate the influence of bionic structural design on energy dissipation properties. With low levels of deformation (5%, 10%, and 15%), PU film exhibited predominantly elastic behavior. It stored elastic energy during compression and fully recovered upon unloading, as shown in Figure 3c. As strain increased, PU polymer chains tended to rearrange and untangle, which resulted in irreversible plastic deformation, leading to dissipation of energy.^[^ ^44^ ^]^ The normalized dissipated energy became noticeable at 50% strain, reaching 0.422 MJ m^−3^ (Figure 3d).

**Figure 4:**
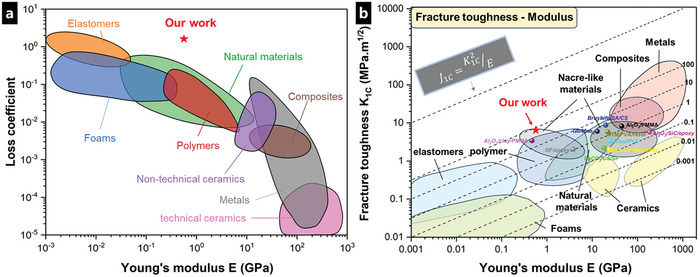
Mechanical performance comparison chart. a) Ashby plot displaying the loss coefficient and Young's modulus of the APU composites in our work (red pentagram) compared to engineering materials. b) Ashby plot displaying the Fracture toughness and Young's modulus of the APU composites in our work (red pentagram) compared to nacre‐like composites (balls with different colors) and other classes of materials.

On the other hand, APU composites displayed distinct viscoelastic behavior. The compression curve at 5% and 10% deformations presented partial deviation due to sample differences. At 50% compressed deformation, APU exhibited a maximum normalized dissipated energy of 8.557 MJ m^−3^, which was nearly 20 times that of PU. Moreover, the loss coefficient tan δ of APU films reached up to 1.6, greatly surpassing PU films (0.25) (Figure 3e). Hence, APU films could provide effective protection for brittle glass substrates when the car went over (Figure S3 and Movie S1, Supporting Information). To highlight the loss coefficient of the APU composites with that of engineering materials,^[^ ^19^ ^]^ we display our experimental data on an Ashby plot (**Figure** **4**a). The overall quasi‐static mechanical properties of APU composites in the application of energy absorption were promising (Figure 3f).

A deeper understanding of how the bionic optimized structure works for quasi‐static energy dissipation was acquired via micro‐CT and SEM analysis of the compressed samples. Figure 3g–i shows the reconstructed 3D structure of APU composites compressed at different strains, while Figure 3k–m presents the corresponding 2D cross sections. Before compression, the Al_2_O_3_ layers were uniform with certain porosity. Upon compression, a reduction in thickness and the emergence of inner‐layer cracks in the porous Al_2_O_3_ layers occurred (Figure 3h,i). Combined with SEM micrographs (Figure S4, Supporting Information), the Al_2_O_3_ nanoparticles densified under compressive forces, and nanopores started disappearing. It was inferred that nanoparticles migrated and flowed to fill the nanopores during compression processes, leading to significant irreversible deformation upon unloading. In addition, multiple cracks occurred in porous Al_2_O_3_ layers after a 15% compression (Figure 3l). The cracks propagated and expanded into substantial gaps at a 60% compressive strain, as shown in Figure 3m. The movement of nanoparticles and new surfaces generated by crack propagation contributed to outstanding static energy absorption.

The toughness of APU composites was estimated by compact tension fracture toughness testing, according to ASTM E1820. We measured a reliable *J*‐integral value for the crack initiation of APU composites at ≈54.13 kJ m^−2^ in our three tested samples (Figure S5, Supporting Information). The fracture toughness (*K_IC_*) can be given through the opening mode *J/K* equivalence relationship. Following Ashby's plot of the comparison of toughness and modulus, APU composites displayed superior fracture toughness than certain nacre‐like materials^[^ ^34^, ^42^, ^45^, ^46^, ^47^, ^48^, ^49^, ^50^, ^51^, ^52^, ^53^ ^]^ and some engineering materials^[^ ^54^ ^]^ (Figure 4b; Table S1, Supporting Information), which could be attributed to the ingenious hierarchical structural design.

### 2.2 Impact Protection Capabilities of Bionic Composites

**Figure 5:**
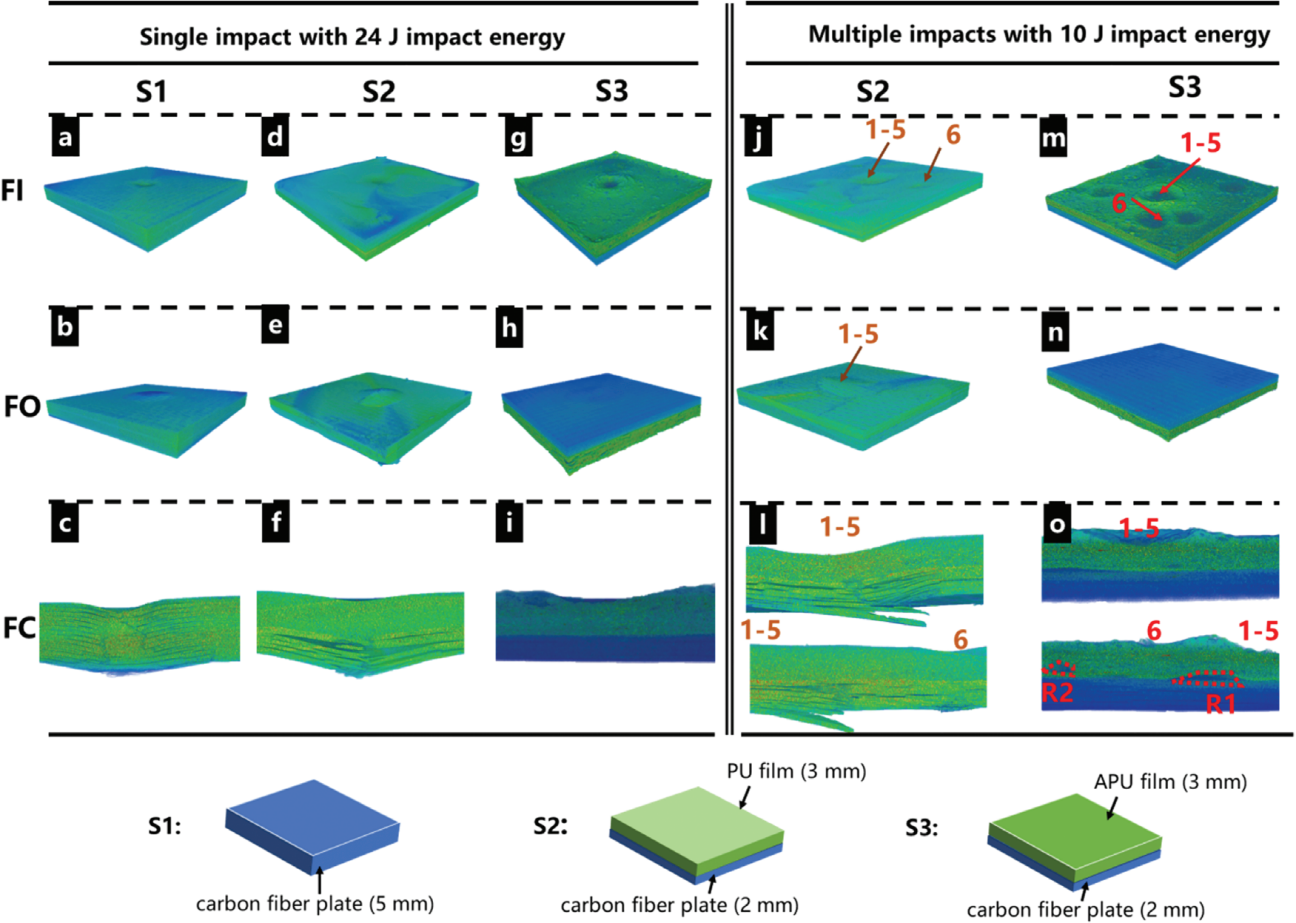
Investigation of dynamic impact mechanical properties. a‐i) Micro‐CT image of impact face (FI), opposite face (FO), and cross–sectional face (FC) of S1, S2, and S3 after signal impact with 24 J impact energy. j–o) Micro‐CT image of FI, FO, and FC of S2 and S3 after multiple impacts.

To illustrate the effect of bioinspired multiscale structural designs on impact protection capabilities, the prepared APU composite films were applied onto carbon fiber‐reinforced plastic (CFRP) plates. Three types of samples were fabricated: as shown in **Figure** **5**, sample 3 (S3) consisted of 2 mm carbon fiber plates with 3 mm APU films, while sample 2 (S2) was prepared by substituting the APU film in S3 with a PU film, and sample 1 (S1) was 5 mm‐thick CFRP plate as control. Drop‐tower impact tests were performed on these samples. The damaged areas on the carbon fiber plates were examined to evaluate the impact protection capabilities of these films. As expected, APU films exhibited significantly superior impact resistance.

The impact tests with varying impact energies were utilized to investigate the maximum impact tolerance. No obvious damage occurred after 24 J impact in the APU‐coated sample (S3), while the carbon fiber plate coated with PU film (S2) and the carbon fiber plate control (S1) showed delamination and fiber breakage at ≈18 J. A ≈33% increase in energy absorption was noticed in APU‐coated samples compared with PU films. According to ASTM D7136,^[^ ^55^ ^]^ the normalized impact energies of APU composites are 245 kJ m^−2^, surpassing certain biomaterials and fiber composites reported in the literature^[^ ^56^, ^57^ ^]^ (Figure S6, Supporting Information).

Surface and internal damages of the samples after 24 J impacts were further investigated using micro‐CT (Figure 5a–i). Specifically, APU samples (S3) exhibited a pronounced plastic deformation on its impact face, whose deformation areas surpassed the additional two samples (Figure 5g,h). While the opposite face of S3 displayed minimal damage. In contrast, control (S1) and PU samples (S2) appeared to have significant deformation and damage on their opposite faces (Figure 5a–e). The cross–sectional face images of internal S3 revealed conspicuous plastic deformation in the APU composite region, and the 2‐millimeter‐thick carbon fiber plate remained remarkably intact (Figure 5i). However, S1 and S2 exhibited significant damage in the carbon fiber plates (Figure 5c,f).

In addition to a single impact, the efficacy of the bioinspired APU films was tested under multiple impacts. We adopted a series of impact tests with 10 J of impact energy, encompassing a total of 10 impact processes. Note that the carbon fiber plates of both S2 and S3 displayed no obvious damage after a single impact with 10 J impact energy (Figure S7, Supporting Information). For multiple impacts, the first 5 impacts were targeted at the same location, while the subsequent 5 were administered at random points. Subjected to the first 5 consecutive impacts at the same location, the PU sample (S2) displayed noticeable damage to the underlying carbon fiber plate (Figure 5j–l). Severe delamination and fiber breakage were observed. Remarkably, for the APU sample (S3), minimal deformation was observed in its underlying carbon fiber plate after 10 consecutive impacts (Figure 5m,n), leaving the pits of the APU surface layer. The interface detachment and the visible voids (R_1_, R_2_) of APU films occurred in Figure 4o. These surface and inner damages within APU films significantly enhanced impact energy absorption, which was not observed in PU films. In conclusion, APU films could provide superior protection for carbon fiber plate substrate from multiple impacts.

Practical applications often involve different substrates and curved surfaces. To address these requirements, we designed several pertinent experiments. Steel and glass substrates commonly used in practical applications were also chosen for impact tests. In identical testing conditions, APU films exhibited superior impact resistance compared to PU films (Figure S8, Supporting Information). For instance, glass plates with APU films remained unscathed under a 5 J impact, while those with PU films were broken into multiple fragments (Movie S2, Supporting Information).

Similarly, steel sheets with APU film exhibited less deformation when compared to their PU film counterparts. The resulting depth of the crater was 1.2 mm, decreasing 29.4% compared to that of PU film. On the other hand, impact tests using sample vials with and without the APU film were employed to substantiate the effectiveness of APU films on the curved surface. After impact with 0.5 J impact energy, the control vials were fractured completely, while the vials with APU films can withstand impact energy of up to 1.2 J (Figure S9, Movies S3 and S4, Supporting Information). In the meantime, sample vials protected with commercial 3M W8607 (or Tesa 52994) polyurethane protective tape were already broken (Movie S5, Supporting Information).

### 2.3 Multiscale Energy Dissipation Mechanisms

**Figure 6:**
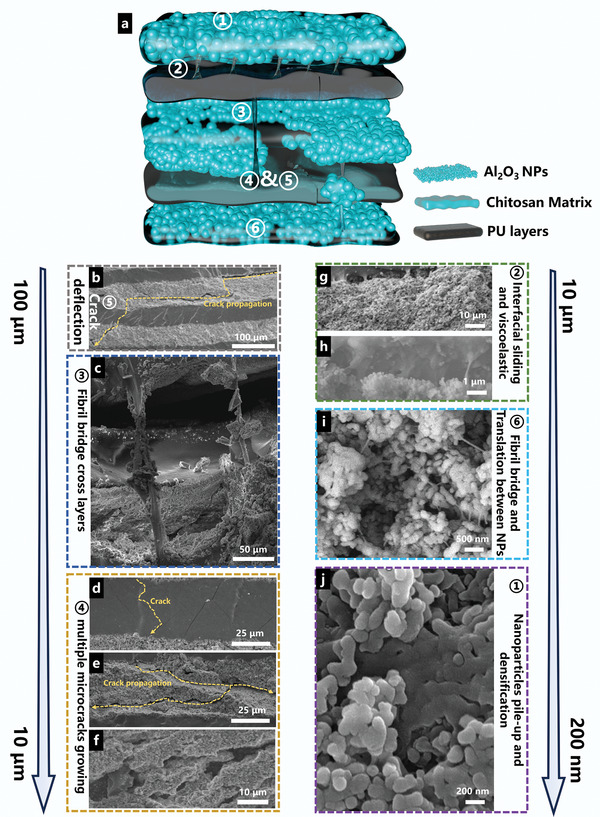
Multiscale energy absorption mechanisms of APU composites. a) A schematic illustration comprising structural changes after impact. b) SEM image of crack deflection between soft layer and hard layer. c) SEM image of fibril bridge cross layers. d‐e) SEM image of multiple microcracks growing g,h) SEM images of interfacial sliding between adjacent layers, and viscous resistance limited excessive adjacent layers mobility. i) SEM image of fibril bridge and translation between nanoparticles. j) SEM image of nanoparticles pile‐up and densification.

Based on the multiscale energy absorption mechanisms observed in mantis shrimp and mollusk shells, we have conducted to elucidate the outstanding impact‐resistance properties of the APU film. A mechanistic schematic, derived from a comprehensive analysis of the SEM figures after impact, is presented in **Figure** **6**. Specifically, the small blue spheres represented Al_2_O_3_ nanoparticles, while the blue boxes indicated the chitosan network connecting these nanoparticles, collectively forming the rigid layer. The gray boxes depicted the soft PU layer. Subsequently, a five‐layer model with interfacial interactions was established to visually convey the structural changes after impact and detailed analysis of structure‐performance relationships from the micro‐ to the nanoscale (Figure 6a). After impact, the surface layer underwent macroscopically visible plastic deformation, where the original nanoparticles in the region of deformation were forced to move and pile up at the edges of the depressions, similar to the energy absorption of mantis shrimp impact surface.

Additionally, the squeezing between layers leads to the densification of hard Al_2_O_3_ layers, consistent with compression tests of APU composites (Figure 6j). Interfacial sliding and interlayer fiber bridging were also observed, akin to nacre (Figure 6g,h). Furthermore, fiber bridge cross layers were also presented with the extracted nanoparticle agglomerates (Figure 6c). In addition, the flexible PU layer was deformed during the impact, resulting in a series of diagonal shear bands (Figure 6d). Weak interfaces between the hard layer and soft layer (or nanoparticles and chitosan matrix) led to crack deflection and the growth of multiple microcracks (Figure 6b,e,f), which was crucial for impact energy absorption. At the nanoscale, relative movements among adjacent particles during impact led to the formation of fiber bridges between nanoparticles (Figure 6h). These multiscale energy absorption mechanisms, ranging from macroscopic to microscopic scales, synergistically enhanced the impact‐resistance performance of APU films.

**Figure 7:**
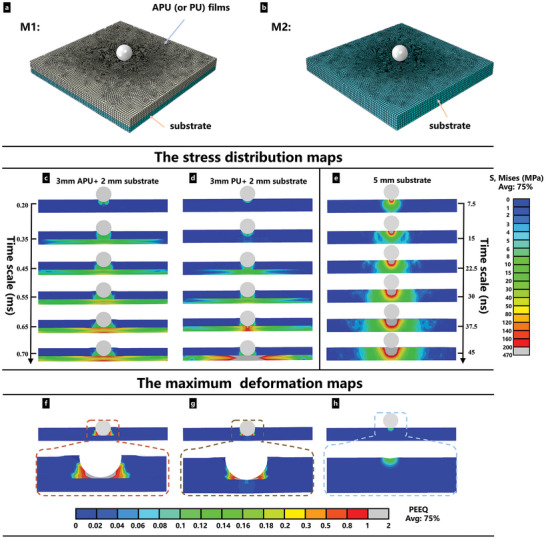
Finite element simulations with different models, force (or deformation) increases with the color changing from top to bottom (or left to right). a, b) The model of M1 and M2 using in FEA. c,d) The stress distributions of different models under impact as a function of time. Significant smaller stresses are shown in the steel substrate under the protection of APU film in (c). f–h) The maximum plastic strains in different models. Large plastic deformation of steel substrate under impacts is noticed in (g) and (h). In models with APU film (f), the deformation is limited in the APU region, and no obvious plastic strain is observed in the steel substrate.

The protection role of APU films was further validated via FEA simulations through ABAQUS. Similar to the drop tower impact tests, the APU or PU films (3 mm) were attached to a 2 mm thick hard solid substrate (model M1 in **Figure** **7**a). Steel was set as the substrate material, as an example. As a control, a pure steel substrate model with 5 mm thickness was created (M2, Figure 7b). In the simulation, a steel ball with 1000 g was set as a projectile with an initial impact speed of 5 m s^−1^ when hitting the target substrates. Based on explicit dynamics analysis, the stress distributions of these models under impacts at different periods are shown in Figure 7c–e. Due to the high stiffness, the impact last for a relatively short time period in M2 compared to M1. It was noticed that the film in M1 was effective in mitigating the stress transmitted to the underlying 2 mm substrate. The APU films offered better protection for the substrate since significantly lower stress levels and plastic deformations occurred in the underlying 2 mm substrate compared to PU films (Figure 7f–h). Large deformation in the APU layer absorbed a substantial amount of impact energy. Moreover, the stress level and deformation increased with the enhanced impact speed and energy (Figure S10, Supporting Information). These findings are consistent with the experimental results, confirming the effectiveness of the APU composites in providing better protection.

## 3 Conclusions and Outlook

Inspired by the mantis shrimp and mollusk shells, a novel biomimetic design of composite film to achieve superior protection of various substrates from compression and multiple impacts was proposed. The APU composite film exhibits alternating soft and hard layers, in which hard layers comprise high‐content (≈90 wt.%) Al_2_O_3_ nanoparticles closely packed and glued together by chitosan macromolecules. The resulting energy absorption under quasi‐static compression is almost 20 times higher than commercial PU films. Under high strain rate impacts, nanoparticle movement, and densification, plastic deformation of the chitosan molecules is the main energy dissipation mechanism in the rigid layer, which is similar to the mantis shrimp dactyl club. The alternating hard and soft layer structure leads to interfacial sliding and crack deflection between adjacent layers, akin to nacre. These multiscale energy absorption mechanisms contribute to the superior impact‐resistance performance of APU composites, whose normalized impact energy surpasses that of PU by 1.33 times. More interestingly, the APU composite maintains its impact resistance even under repeated impacts and on curved surfaces. The biomimetic APU composites effectively inherit the superior results of natural selection and provide an extended design approach for the development of highly advanced impact‐resistant materials suitable for complex application environments. The relative thickness of the layers, concentration, and shape of particles can be optimized to meet different loading environments. The energy dissipation at atomic and molecular scales are not covered in this research yet, which are supposed to play important roles in the overall performance. Integrating heterogeneous structures at different length scales to improve energy dissipation is one of the main goals of the future study.

## 4 Experimental Section

### Preparation of APU Composites

First, the highly content Al_2_O_3_ slurry was prepared using a mixture of 150 nm alumina nanoparticles, chitosan, and a blend solvent of 1 M acetic acid and alcohol (1:1 ratio). These materials and reagents were sourced from Aladdin Reagent. In detail, 10.8 g of Al_2_O_3_ nanoparticles were added to 40 mL of the blend solvent with 30 min ultrasound dispersion. Subsequently, 1 g of chitosan was introduced into the resulting mixture, and it was gently mechanically stirred for 10 min, then left undisturbed for 8 h. The resulting mixture was then processed in a mixer, rotating at 1500 rpm for 1 minute to form the final slurry.

To further construct the lamellar architecture, the above highly content Al_2_O_3_ slurry and 40 wt.% aqueous polyurethane emulsion (Shanghai Macklin Biochemical Co., Ltd) were alternately coated by Spin coater or blade coater. Release agents were sprayed onto a clean substrate. The slurry was uniformly spin‐coated at 500 rpm for 1 min on this substrate. Subsequently, the hard Al_2_O_3_ layer was formed at 50 °C as the new substrate. Next, the soft PU layer was applied to the hard layer using spin‐coating at 3000 rpm and then dried. Following the above steps, APU composites were prepared layer‐by‐layer with varying thicknesses. The large‐scale APU films were prepared by blade coating.

### Preparation of Test Samples

A layer of aqueous polyurethane emulsion was spin‐coated on the carbon fiber plate. Subsequently, the APU or PU films were placed on top of it. Then, all the samples were heated at 80 °C for 5 min, resulting in the formation of samples S3 and S2. The sample vials with APU were formed by a similar process. Aqueous polyurethane emulsion was spin‐coated on APU composites. This APU wrapped around the sample vials heated at 80 °C for 5 min. The APU or PU films of S3 and S2 were 3 mm thick. The APU or PU films used in glass plates under 5 J impact were 1 mm thick. The APU or PU films used in steel sheets under 10 J impact were 1 mm thick. The APU films used in sample vials were 0.8 mm thick.

### Sample Characterizations

The mass fraction of Al_2_O_3_ in the hard layers was measured by Pyris1 TGA (PerkinElmer). The temperature range of TGA was 30 to 800 °C, while the heating rate was 10 °C min^−1^. The 3D scanning structure was measured by µCT 100 cabinet micro‐CT scanner (SCANCO), with a scanning accuracy of 24.5 µm. The microstructure of APU composites was observed by FSEM (JSM‐7600F, JEOL) and TEM (JEM‐2100, JEOL). The impact processes were captured by mobile phones.

### Mechanical Testing

Static tensile and compression tests were performed on a Multi‐material mechanical property tester (C45.105EY, MTS). Tensile and compression samples were dumbbell and square‐shaped samples, respectively. To minimize experimental error, multiple samples were prepared for testing, and the resulting curves represent the average values for each sample. The quasi‐static tensile experiment employed a large deformation extensometer with a strain rate of 0.005 s^−1^. When subjected to compression, the strain rate was 0.006 s^−1^: the storage modulus and tan δ acquired from DMA testing (Diamond DMA, PerkinElmer). The impact tests, both signal and multiple impacts, were conducted using BGD305 Heavy‐Duty Impact Tester (Biuged Instruments Co., Ltd). In the case of a single impact test, a 2 kg drop hammer (12.7 mm in diameter) was used. The drop heights for the single impact tests were 120 cm, resulting in 24 J of energy with a velocity of 4.9 m ^−1^s, 100 cm for 20 J of energy, and so on. For the multiple impact tests, a 1 kg drop hammer (12.7 mm in diameter) was used, and the drop height was set at 100 cm with a velocity of 4.5 m ^−1^s. For the impact tests of sample vials with APU films, the TG‐921H impact tester was utilized, which employed a 200 g steel ball.

### Fracture Toughness Testing

Compact tension fracture toughness testing used an Electromechanical universal testing machine (UTM2022, Shenzhen Suns Technology Stock Co., Ltd). The test was performed by applying equal and opposite forces in the specimen with a displacement rate of 1 mm min^−1^, to propagate the initial sharp crack. The thickness (*B*) of tested samples was ≈1 mm. The in‐plane dimensions along the transversal direction (*W*) were ≈50 mm. The in‐plane dimension along the longitudinal direction (*C*) was between 50 and 60 mm. To provide a pre‐notch, a rough cut and then a sharpened cut were used. The total length of pre‐notch (*a*) was ≈25 mm. An anti‐buckling guide plate prevented the sample from bending out of plane during the test, shown in Figure S5a (Supporting Information).

The *J*‐integral area (*J*) contains elastic part (*J*_el_) and plastic parts (*J*_pl_) under the load versus load‐line displacement curve. *J* was calculated using the following equations:

(1) $$J=\frac{\eta A_{T}}{Bb}$$

where *A*_T_ was the total area (elastic plus plastic), *b* was ahead of the crack tip (*w* − *a*), η was the specimen geometry factor (given by η  =  2 + 0.522*b*/*W*).

Fracture toughness (*K*_1*C*_) was calculated using the following equations:

(2)

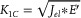

Where *E*′ =  *E*(1 − *v* ^2^), *E* represents Young's modulus, and *v* represents Poisson's ratio.

### Normalized Impact Energy Calculation

These calculations were according to the ASTM standard D7136/ D7136 M‐07. The normalized impact energy was calculated according to:

(3) $$E_{n}=mgh/d_{s}t$$

Where *E_n_* was the normalized impact energy, *m* was the mass of the impactor, *g* was the gravitational constant, *h* was the height from which the impactor was dropped, *d_s_* was the cover plate aperture diameter, and *t* was the thickness of the sample.

### Finite Element Simulations

Using the commercial software ABAQUS, the construction of a model was undertaken and finite element simulations of an impact process were conducted. To accurately replicate experimental conditions, three models were established, each with a total thickness of 5 mm and dimensions of 50 mm × 50 mm. The response of the substrate was simulated using a linear elastic model with Young's modulus (*E*) set to 150 000 MPa and Poisson's ratio (ν) to 0.3. To mimic the experimental situations, APU (or PU) and the substrate were set to have a rigid contact, and no separation was allowed after contact. Hyperelastic Marlow models were applied to simulate the mechanical behaviors of APU and PU films based on the stress‐strain curves obtained in the uniaxial compression tests. Dynamic explicit analysis was employed for computation. The smallest time step was 0.015 ms. The models were meshed using ABAQUS solid elements (C3D8R). The number of nodes of the finite element model was ≈120 000. The number of elements was ≈100 000.

### Statistical Analysis

All the data were obtained from at least three independent experiments, and the experimental data were presented as the mean value with standard deviation. All the statistical analyses were performed using origin software.

## Conflict of Interest

The authors declare no conflict of interest.

## Supporting information

Supporting Information

Supplemental Movie 1

Supplemental Movie 2

Supplemental Movie 3

Supplemental Movie 4

Supplemental Movie 5

## Data Availability

The data that support the findings of this study are available from the corresponding author upon reasonable request.;
