# Supplementary material for: A Bioinspired Design of Protective Al2O3/Polyurethane Hierarchical Composite Film Through Layer‐By‐Layer Deposition
Source: Adv Sci (Weinh). 2024 May 20;11(28):2402940. doi: 10.1002/advs.202402940 (PMC11267295; doi:10.1002/advs.202402940)
Supplement: Supplementary file 1 — Supporting Information [file ADVS-11-2402940-s001.pdf]

ADVANCED SCIENCE
Open Access

# Supporting Information

for *Adv. Sci.*, DOI 10.1002/advs.202402940

A Bioinspired Design of Protective $Al_2O_3$/Polyurethane Hierarchical Composite Film Through Layer-By-Layer Deposition

*Jiaming Zhong, Zhixiong Wen, Yibo Wu, Hao Luo, Guodong Liu, Jianqiao Hu, Hengxu Song, Tao Wang, Xudong Liang, Helezi Zhou, Wei Huang* and Huamin Zhou**

**Supporting Information**

# A bioinspired design of protective $Al_2O_3$/polyurethane hierarchical composite film through layer-by-layer deposition

Jiaming Zhong[1#], Zhixiong Wen[1#], Yibo Wu[2], Hao Luo[2], Guodong Liu[2], Jianqiao Hu[3], Hengxu Song[3,4], Tao Wang[5], Xudong Liang[6], Helezi Zhou[1], Wei Huang[1*], Huamin Zhou[1*]

1. State Key Laboratory of Materials Processing and Die & Mould Technology, School of Materials Science and Engineering, Huazhong University of Science and Technology, Wuhan, 430074, China
2. Luoyang Ship Material Research Institute, Luoyang 471023, China
3. LNM, Institute of Mechanics, Chinese Academy of Sciences, Beijing 100190, China
4. School of Engineering Science, University of Chinese Academy of Sciences, Beijing 100049, China
5. National Key Laboratory of Explosion Science and Safety Protection, Beijing Institute of Technology, Beijing 100081, China
6. School of Science, Harbin Institute of Technology (Shenzhen), Shenzhen, 518055, China

# These authors contributed equally to this work.

*Corresponding author: mse_huangw@hust.edu.cn; hmzhou@hust.edu.cn

# Content

**Additional file 1: Figures** ........................................ 1

**Figure S1**. TGA curve revealing the mass fraction of $Al_2O_3$ nanoparticles in the hard layer. ........................................ 1

**Figure S2**. Shore hardness. ........................................ 2

**Figure S3**. Glass plate (right) and glass plate with APU films (left) after the car go-through test. ........................................ 3

**Figure S4**. SEM images showing the nanoparticle connections. ........................................ 4

**Figure S5**. Investigation of Fracture Toughness ........................................ 5

**Figure S6**. Comparison of normalized impact energy of this work and reported papers. ........................................ 6

**Figure S7**. Investigation of impact damage during 10 J single impact. ........................................ 7

**Figure S8**. APU films offering enhanced protection for steel and glass substrates in comparison to PU films. ........................................ 8

**Figure S9**. Studying the protection performance of APU composites during curved surface impact.. ........................................ 9

**Figure S10**. Finite element simulations with different speeds.. ........................................ 10

**Additional file 1: Table** ........................................ 11

**Table S1** | Summary of previously reported nacre-like composites ........................................ 11

**Additional file 1: Figures**

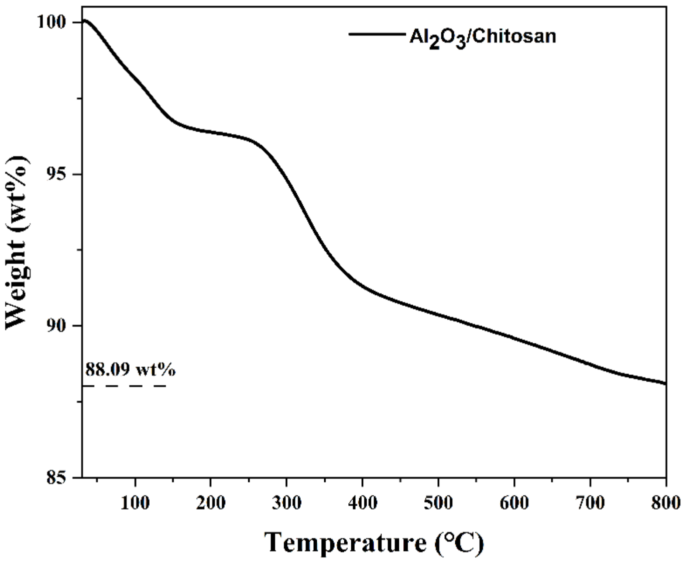


**Figure S1**. TGA curve revealing the mass fraction of $Al_2O_3$ nanoparticles in the hard layer.

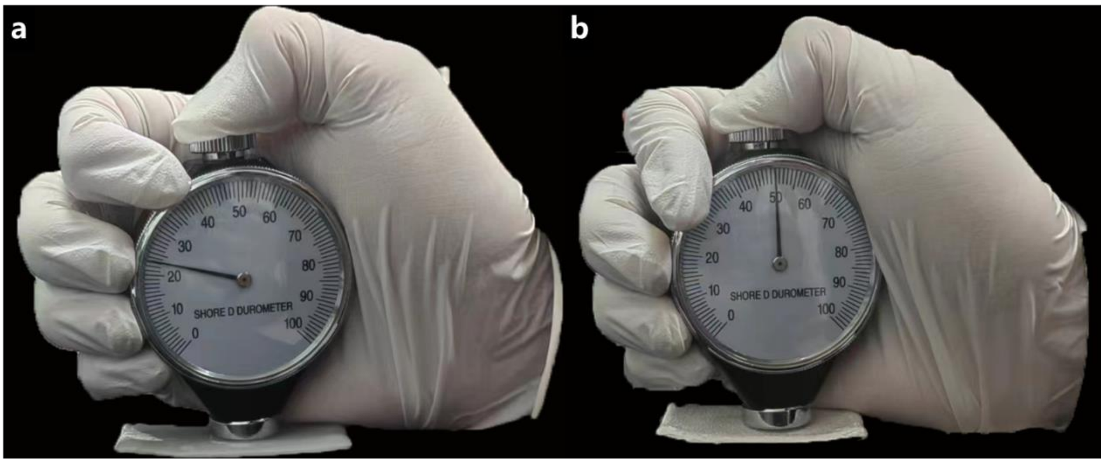


**Figure S2**. Shore hardness of (a) PU composites and (b) APU.

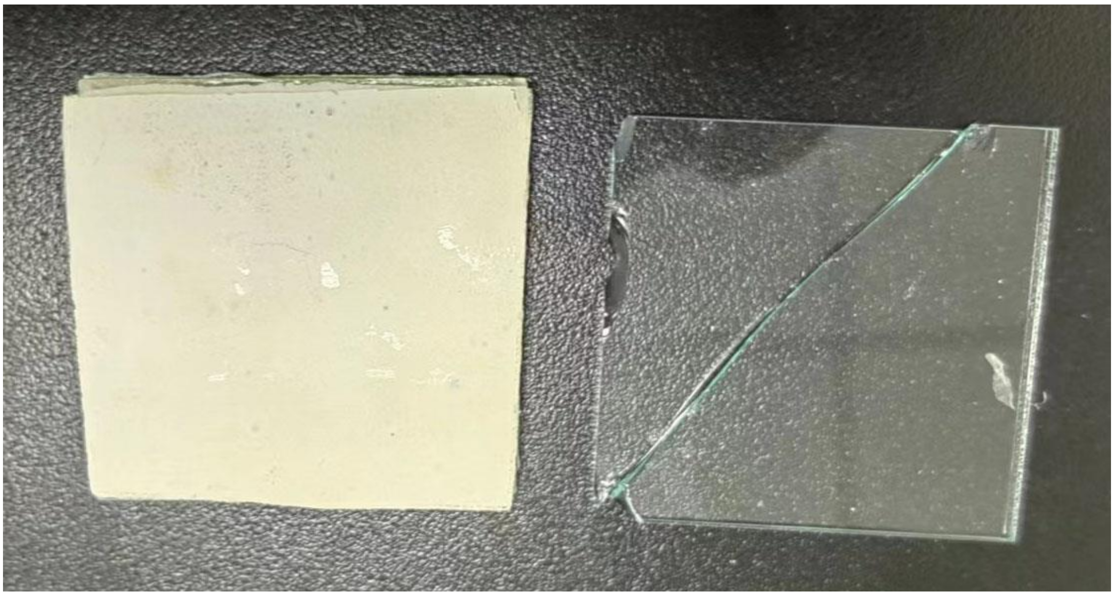

**Figure S3**. Glass plate (right) and glass plate with APU films (left) after the car go-through test. The weight of the car is ~2300 kg.

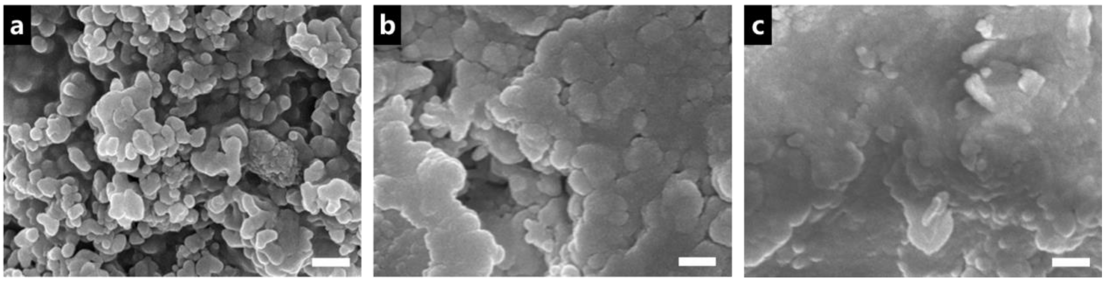


**Figure S4**. SEM images showing the nanoparticle connections. a) APU film before compression. b) APU film after a 15% compression. c) APU film after a 50% compression. All of the scale bars were 200 nm.

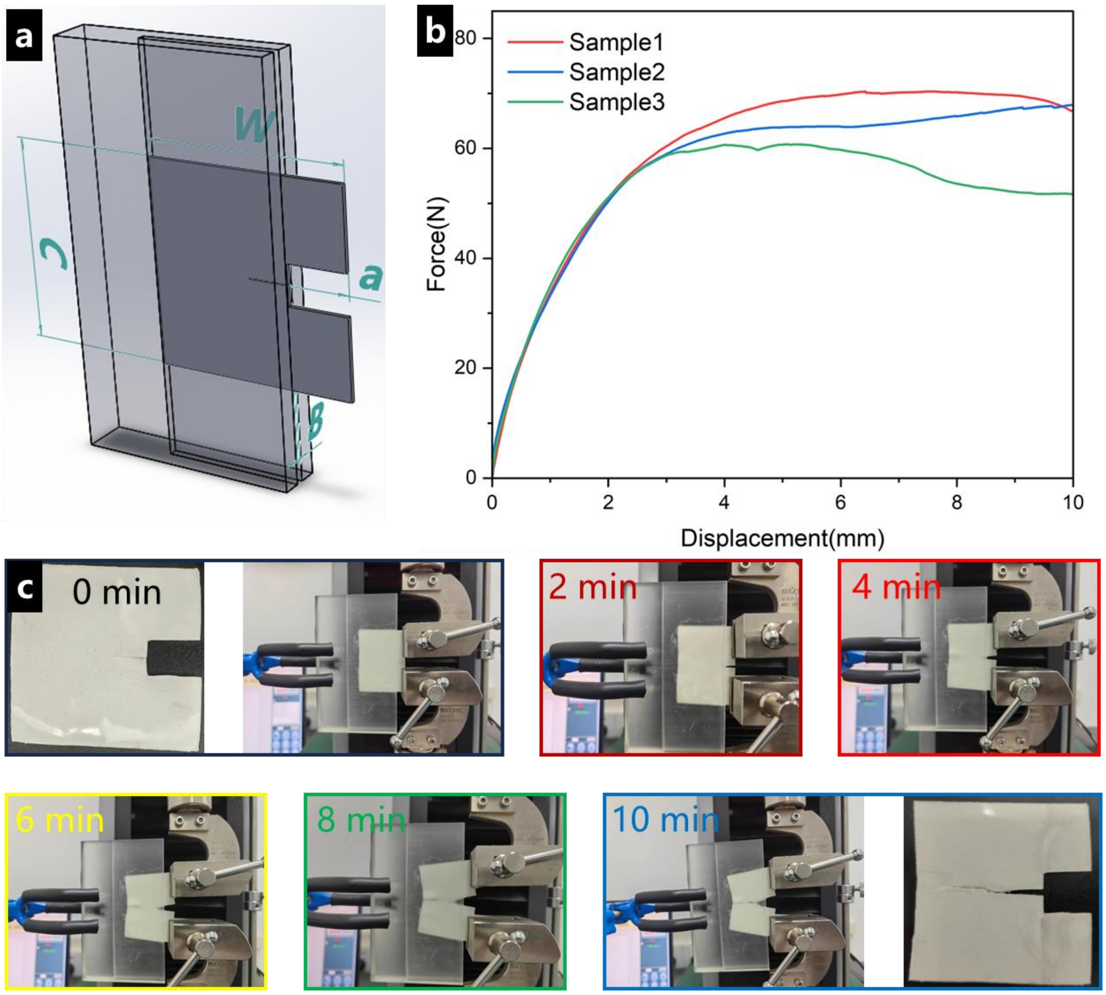


**Figure S5**. Investigation of Fracture Toughness. a) Schematic illustration of tested sample and anti-buckling guide plate. b) Force-Displacement curve from compact tension fracture toughness testing. c) The process of compact tension fracture toughness testing.

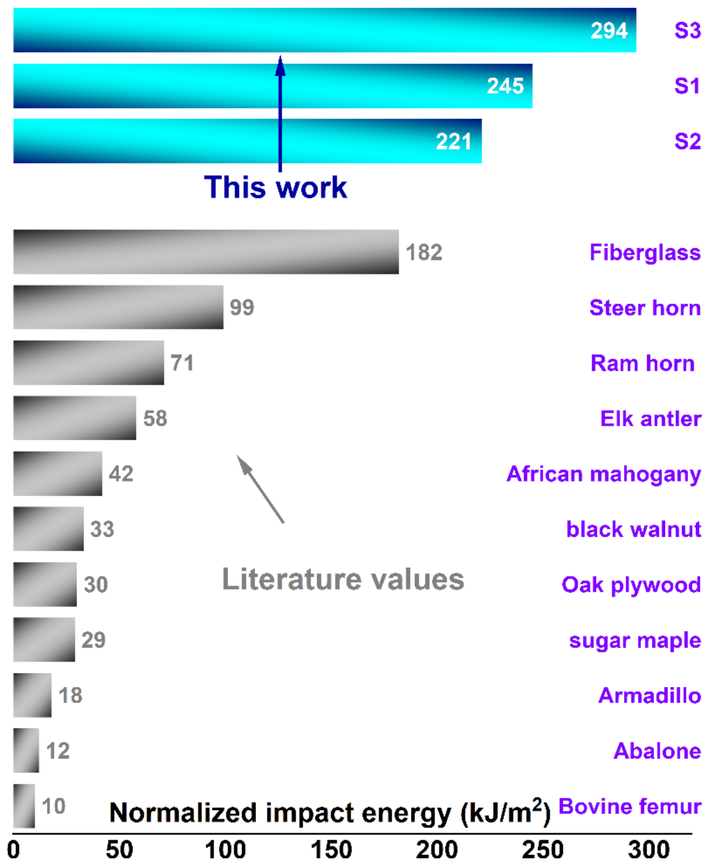


**Figure S6**. Comparison of normalized impact energy of this work and reported papers.

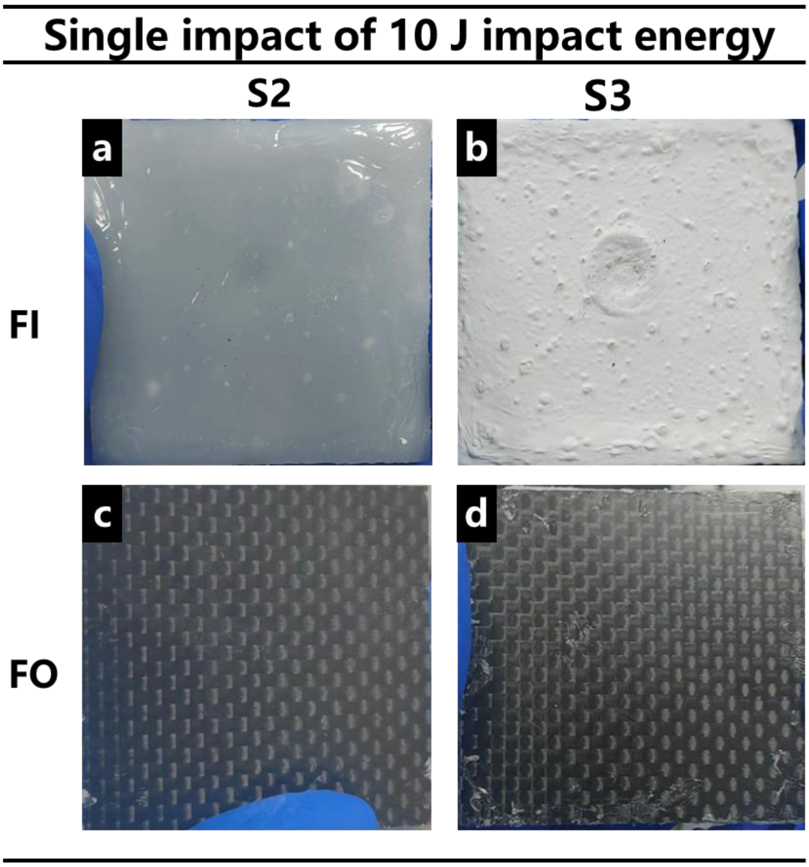


**Figure S7**. Investigation of impact damage during 10 J single impact. a-d) image of impact face (FI) and opposite face (FO) of S2 and S3.

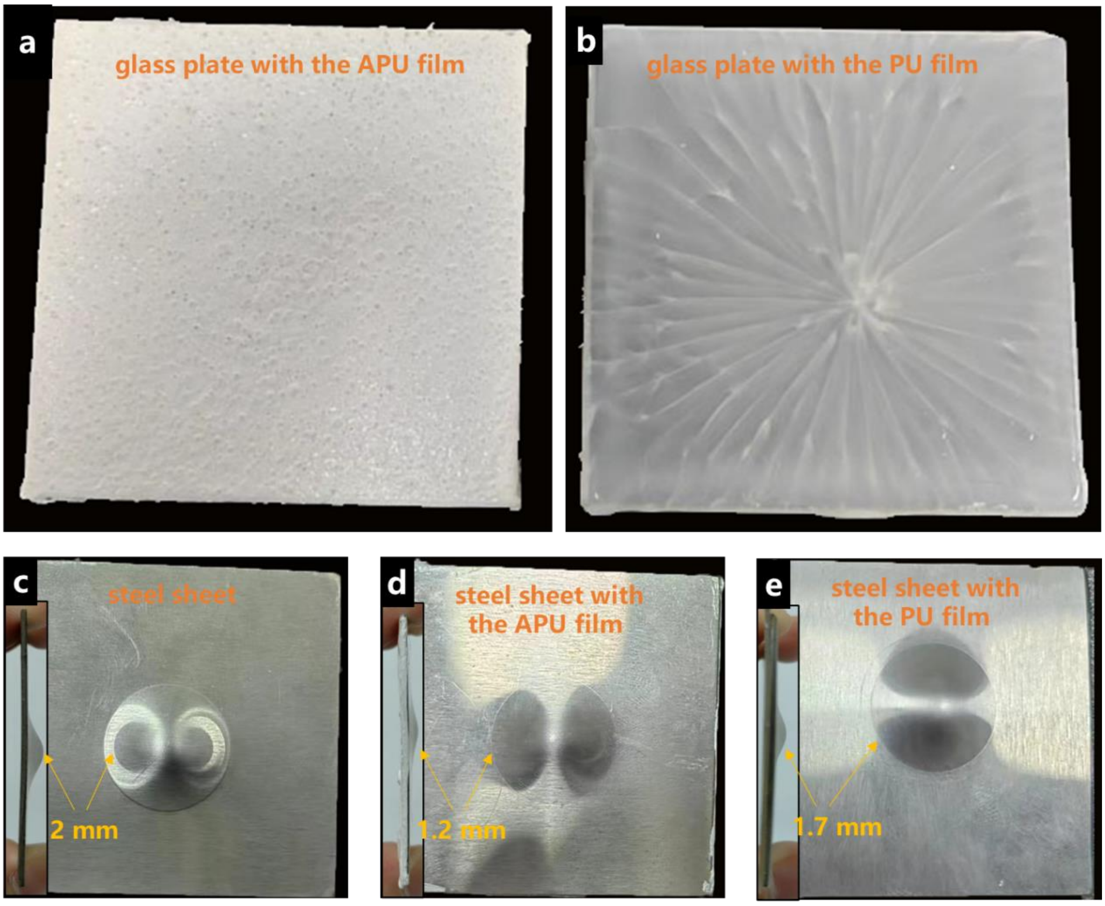


**Figure S8**. APU films offering enhanced protection for steel and glass substrates in comparison to PU films. a,b) Photos of glass plates with the APU (or PU) film after 5 J impact. c,d,e) Photos of steel sheet, steel sheet with the APU film and steel sheet with the PU film after 10 J impact.

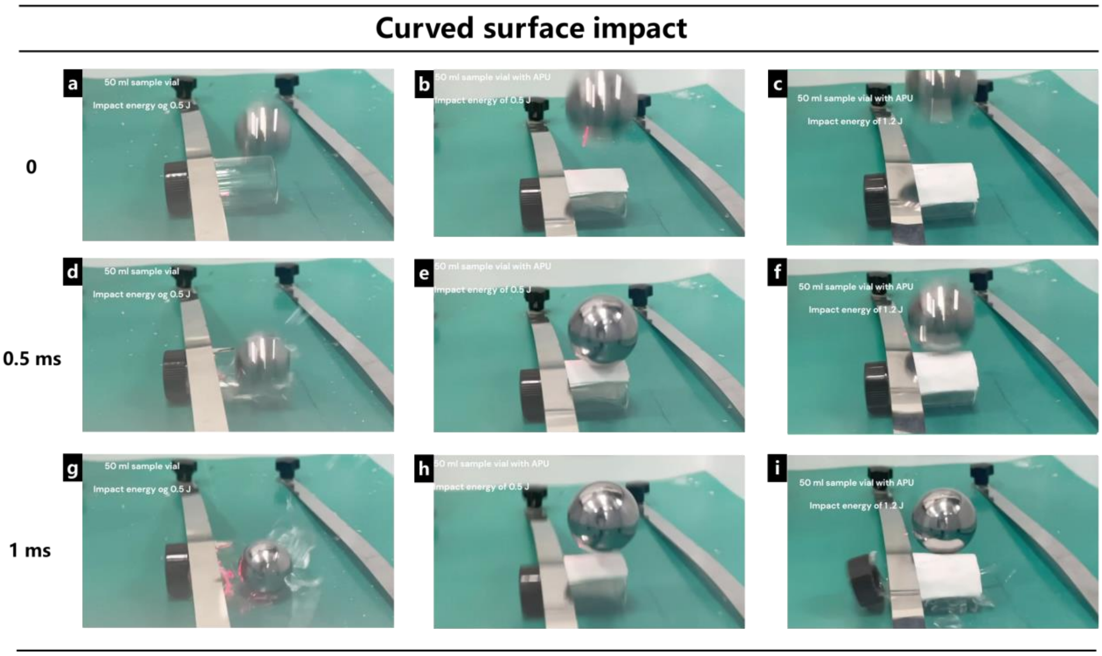


**Figure S9**. Studying the protection performance of APU composites during curved surface impact. a,d,g) the images of the 50 ml sample vial withstanding 0.5 J impact at different times. b,e,h) the images of the 50 ml sample vial with APU composites withstanding 0.5 J impact at different times. c,f,i) the images of the 50 ml sample vial with APU composites withstanding 1.2 J impact with different times.

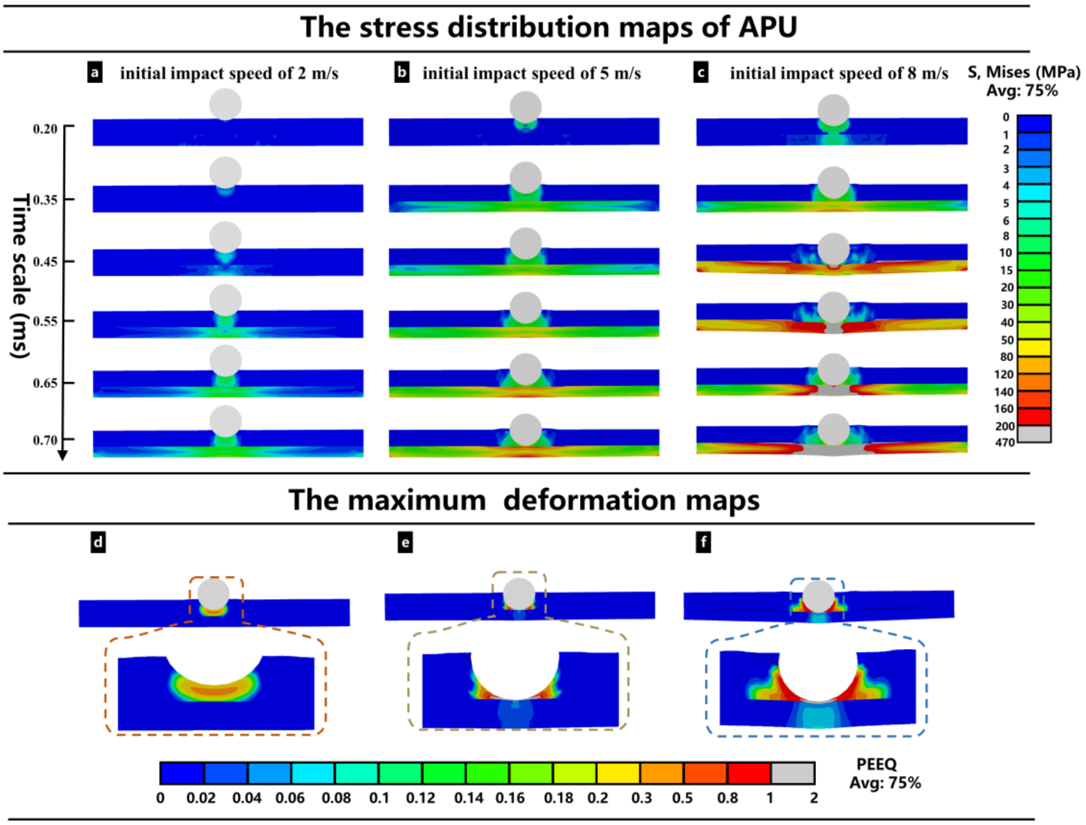


**Figure S10**. Finite element simulations with different speeds. a) the initial impact speed of 2 m/s. b) the initial impact speed of 5 m/s. c) the initial impact speed of 8 m/s. d-f) The maximum plastic strains in different models.

**Additional file 1: Table**

**Table S1** Summary of previously reported nacre-like composites

| composite | modulus E (GPa) | toughness $K_{1C}$ (MPa m$^{1/2}$) | Ref |
|---|---|---|---|
| **$Al_2O_3$/PMMA** | ~45.0 | ~8.2 | Ref.46 |
| **GF/epoxy** | ~3.8 | ~2.0 | Ref.47 |
| **$CaCO_3$/CS/SF** | ~18.3 | ~2.2 | Ref.34 |
| **Brushite/SA/CS** | ~20.0 | ~8.7 | Ref.48 |
| **Clay/PVA** | ~25.0 | ~3.4 | Ref.49 |
| **$Al_2O_3$/SiC/epoxy** | ~186.0 | ~5.7 | Ref.50 |
| **Glass/PMMA/PHN** | ~24.0 | ~2.0 | Ref.51 |
| **MTM/PVA/resol** | ~22.0 | ~5.8 | Ref.52 |
| **G0/$Mno_2$** | ~13.0 | ~5.9 | Ref.53 |
| **$Al_2O_3$/clay/PMMA** | - | ~3.4 | Ref.42 |
